# Increased immunocompetence and network centrality of allogroomer workers suggest a link between individual and social immunity in honeybees

**DOI:** 10.1038/s41598-020-65780-w

**Published:** 2020-06-02

**Authors:** Alessandro Cini, Adele Bordoni, Federico Cappa, Iacopo Petrocelli, Martina Pitzalis, Immacolata Iovinella, Francesca Romana Dani, Stefano Turillazzi, Rita Cervo

**Affiliations:** 1 Dipartimento di Biologia, Università di Firenze, Via Madonna del Piano 6, 50019 Sesto Fiorentino, Firenze, Italy; 2 Centre for Biodiversity and Environment Research, University College London, Gower Street, London, WC1E 6BT UK; 3 CISM, Mass Spectrometry Centre, Università di Firenze, Via U. Schiff 6, 50019 Sesto Fiorentino, Firenze, Italy

**Keywords:** Behavioural ecology, Social evolution

## Abstract

The significant risk of disease transmission has selected for effective immune-defense strategies in insect societies. Division of labour, with individuals specialized in immunity-related tasks, strongly contributes to prevent the spread of diseases. A trade-off, however, may exist between phenotypic specialization to increase task efficiency and maintenance of plasticity to cope with variable colony demands. We investigated the extent of phenotypic specialization associated with a specific task by using allogrooming in the honeybee, *Apis mellifera*, where worker behaviour might lower ectoparasites load. We adopted an integrated approach to characterize the behavioural and physiological phenotype of allogroomers, by analyzing their behavior (both at individual and social network level), their immunocompetence (bacterial clearance tests) and their chemosensory specialization (proteomics of olfactory organs). We found that allogroomers have higher immune capacity compared to control bees, while they do not differ in chemosensory proteomic profiles. Behaviourally, they do not show differences in the tasks performed (other than allogrooming), while they clearly differ in connectivity within the colonial social network, having a higher centrality than control bees. This demonstrates the presence of an immune-specific physiological and social behavioural specialization in individuals involved in a social immunity related task, thus linking individual to social immunity, and it shows how phenotypes may be specialized in the task performed while maintaining an overall plasticity.

## Introduction

Division of labour is defined as the pattern of specialization by cooperative individuals of a social group, which perform different tasks or assume specific roles depending on their morphology (polyphenism) or behaviour (polyethism)^1,2^.

These task specializations often occur with a suite of behavioural and physiological correlates, some of which are specific phenotypic specializations that increase the aptitude, the efficiency and/or decrease the costs of the task performed^3–6^. However, the phenotypic specialization associated with division of labour is expected to be under contrasting selective forces: a colony might benefit from having sets of workers with highly specialized phenotypes, highly efficient and apt to perform the appointed task; at the same time, specializing may limit task flexibility, therefore reducing performance of other tasks when needed^7,8^. Thus, division of labour should show an adequate degree of flexibility to allow the colony to rapidly reallocate its resources in response to the environmental demands^9,10^. Understanding the degree of phenotypic specialization in group of workers performing specific tasks has the potential to unravel the trade-off between phenotypic plasticity and specialization.

Allogrooming^11^ is a behaviour in which a worker uses its mouth parts to remove debris from the body of other colony members. This behaviour, observed in several species of eusocial insects, plays a role in defence against parasites and pathogens^12–15^. In honeybees, allogrooming represents an important resistance mechanism that seems to limit ectoparasites load, especially mites, within colonies^16–20^ and its expression depends on genetic and environmental factors^21,22^. In *Apis cerana*, allogrooming is performed at a high rate and appears to be a particularly effective counter-adaptation against the major worldwide threat for honey bee colonies and apiculture, the parasitic mites *Varroa destructor*^16,17,20,23^. The relatively recent spread of this parasite in many countries heavily impacted on *Apis mellifera* colonies^23^ but effective strategies to deal with this emergency are still lacking^23–25^. Being allogrooming expressed with efficacy against *V. destructor* in *A. cerana*, its characterization in *A. mellifera* may clarify its possible effectiveness to control parasite load at colony level. Despite the potential value that allogrooming could have in maximizing colony resistance to parasites and disease transmission, very scarce attention has been given to the behavioural and physiological specializations of allogrooming at the individual level. Thus, the degree of specialization of individuals performing this task is still unclear and behavioural and physiological correlates of allogroomers are largely unknown.

Here, we investigated the degree of phenotypic specialization of allogroomers in the honeybee *A. mellifera*. Empirical evidence reported in literature about the temporal expression of allogrooming and the degree of behavioural specialization in individuals performing this task is contrasting. According to some authors, in *A. mellifera* this behaviour is temporally restricted from the 1^st^ to the 20^th^ day post-emergence^26^ while other researchers observed workers performing allogrooming during their entire life^27,28^. We thus characterized the temporal and spatial dynamics of allogrooming occurrence. To assess the timing of allogrooming expression, we performed detailed behavioural observations on a large cohort of workers along their lifespan inside the hive (until the onset of foraging activities). We then characterized the spatial occurrence of allogrooming events on the comb surface, in order to test whether they are randomly distributed or clustered in specific areas.

Moreover, we investigated the degree of behavioural specialization of allogroomers, focusing on their individual behavioural profile (i.e. the array of tasks performed) and on the role they play in the colony social network. Indeed, it is not clear to what extent specialization in allogrooming implies a different behavioural repertoire in allogroomers compared to same age range non-grooming nestmates. Previous studies showed that allogroomers are specialized individuals performing the behaviour at a consistently higher frequency compared to other tasks typical of same age range workers^29^, while others reported that this behaviour is unfrequently expressed also by the few individuals performing it, which also carry out all the tasks typical of their age^30^. We thus compared the behavioural repertoire of allogroomers with that of same age range non-grooming bees, predicting that, in case of specialization, allogroomer workers would show a reduced performance of the other in-hive tasks (*prediction 1*). In *A. mellifera* colonies, individuals have been shown to occupy different positions (more or less central, i.e. more or less connected) within the colony social network, according to their caste, age and task^31^, producing a compartmentalized structure that likely reduces disease transmission^32^. If allogroomers are behaviourally specialized, we expect that their position within the colony social network differs from that of same age range non-grooming bees. In particular, since this behaviour is expected to be most advantageous if directed towards many bees within the hive, we might predict allogroomers to be more central in the colony network than same-age range non-grooming bees (*prediction 2*).

It would be advantageous for allogroomers to be able to detect nestmates needing to be groomed. Since many stressors, including pathogens and parasites, alter the odour of workers in *A. mellifera*^33–35^, an enhanced perception of such chemical cues could contribute to a possibly specialized phenotype of allogroomers. Indeed, antennae play a key role in the expression of hygienic behaviours, as recently demonstrated by a transcriptomic study of *Varroa* sensitive hygienic bees^36^. Proteomic investigation also showed that honeybee individuals performing different tasks differ for antennal profile of proteins involved in olfaction, with soluble olfactory proteins, which play a crucial role in the first steps of odour recognition, being differently expressed^37^. Among these proteins, two Odorant Binding Proteins (OBPs) have been reported as biomarkers linked to social immunity and shown to have good affinity towards ligands released by decaying insect corpses^38^. A further soluble olfactory protein belonging to the Chemosensory protein family has been found to be associated with grooming behaviour^39^. In these two studies the authors examined the antennal proteomic profiles of honeybees selected and tested for hygienic behaviours, while, to the best of our knowledge, no study so far directly compared the expression of proteins involved in olfaction of bees performing allogrooming. We thus investigated the expression of olfactory proteins in the antennae, predicting that if allogroomers have any degree of chemosensory specialization, this would reflect in a different antennal proteomic profile (*prediction 3*).

Performing allogrooming is likely to increase the risk for allogroomers to come in contact with pathogens and parasites. Allogroomers would therefore benefit from having an increased immunocompetence compared to same age range workers performing different tasks, in order to cope with a higher risk of infection. We tested this prediction by comparing immunocompetence ability between allogroomers and non-grooming workers of same age range, predicting a higher level of immunocompetence in the formers (*prediction 4*).

**Figure 1:**
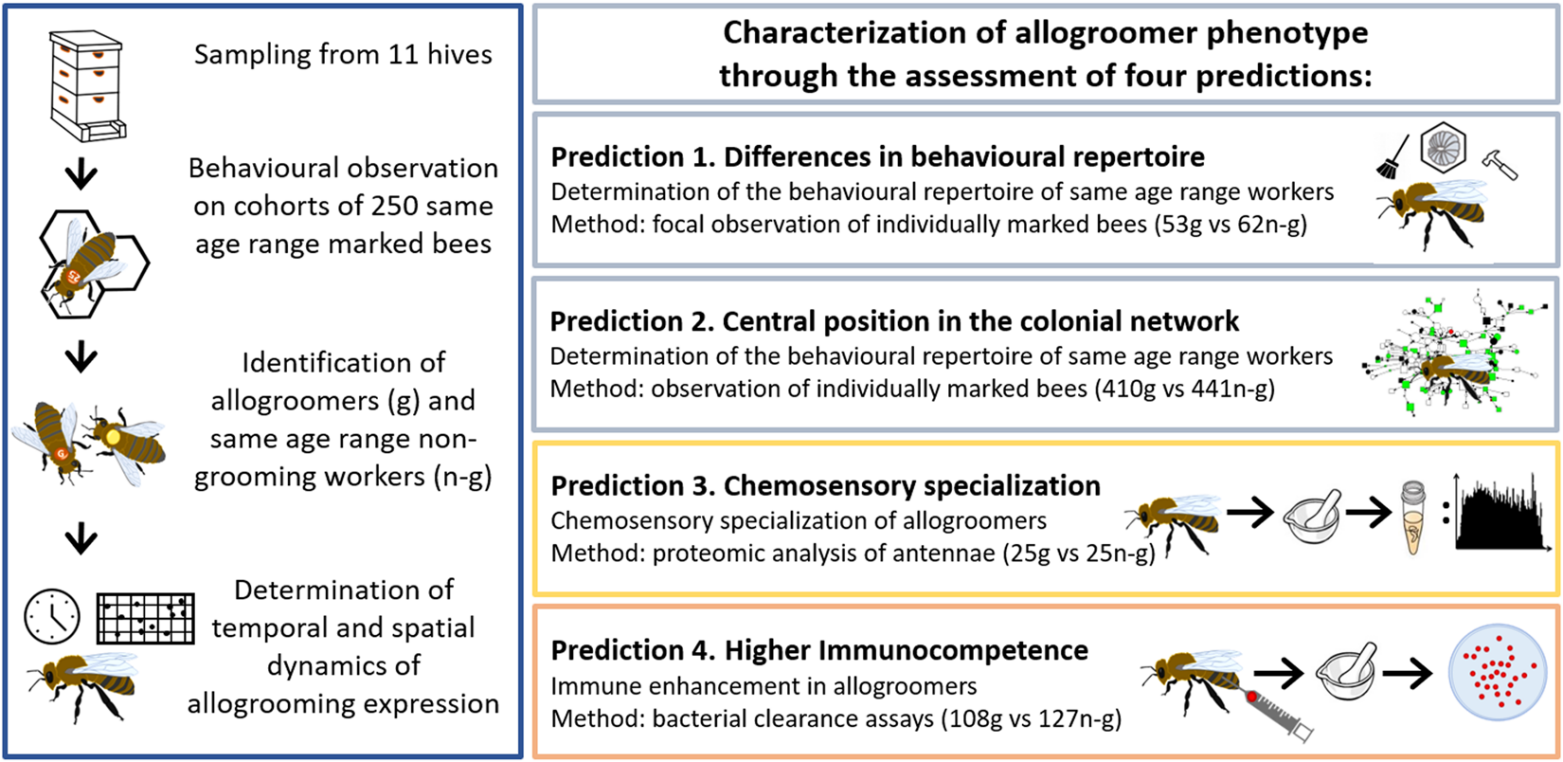
The experimental design of the study, which illustrates sample collection and the predictions tested (g = allogroomers, n-g = non-allogroomers).

Overall, our work characterizes for the first time the allogroomers’ phenotype through an integrated approach which encompasses behavioural observations, proteomics and immuno-assays (Fig. 1).

## Results

### Temporal and spatial dynamics of allogrooming expression

**Figure 2:**
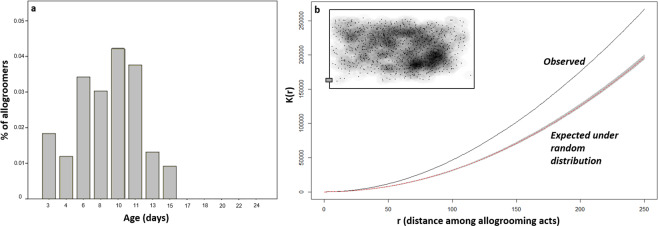
Temporal and spatial dynamics of allogrooming. (**a**) Expression of allogrooming is age-dependent, on average 105 marked bees were observed for each age interval; (**b**) allogrooming is not randomly expressed on the whole comb surface; plot reports the Monte Carlo estimates (MCE) of observed, K(r), vs. expected values of Ripley’s K-function as a function of distance among allogrooming acts (r); solid black line = estimated K(r), dashed line = theoretical K(r) in the setting of complete spatial randomness for the same number of observations, gray-shaded area = estimates of potential variability in K(r) generated by MCE with n = 999 simulations; 1065 events of allogrooming observed in total.

Occurrence of allogrooming clearly varies with bee age (Fig. 2a), occurring within an age-range of 3 to 15 days, and being especially common in the range 6 to 11 days (76% of allogrooming events was observed within this range). The age dependency of this temporal trend is supported by Runs test, which shows significant departure from randomness (Runs test, number of runs = 3, p = 0.013). Furthermore, the fraction of allogroomers that was seen grooming at least once over the total amount of marked bees varies with bees age, showing the same range of 3 to 15 days for grooming expression and with the largest part of allogroomers having an age between 6 and 11 days (78% of allogroomers). Percentage of marked bees that performed at least once allogrooming was 1.50 over the entire observation period and rise up to 4.30 in the peak range (6 to 11 days). The majority of allogroomers (57.1%) was observed performing only once allogrooming.

We observed 1065 allogrooming events in total (respectively 466, 305, 294 on each of the three combs). Spatial distribution of allogrooming strongly deviated from complete randomness, being more clustered than expected (Fig. 2b). In particular, allogrooming events preferentially took place in the area of the comb where brood was present, opposed to the hive entrance and to the dance area.

### Prediction 1. Allogroomers do not show a specific behavioural repertoire

**Figure 3:**
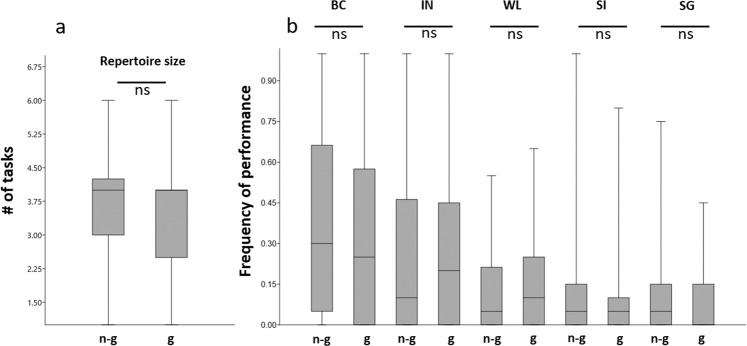
Allogroomers do not show a specific behavioural repertoire. Comparisons between groomers (g, n = 53) and non-groomers (n-g, n = 62) in the repertoire size (**a**) and in the performance rate of five behaviours (**b**) (median and quartiles are represented), for abbreviation and statistic results see Table 1. Only behaviours with average performance rate higher than 0.05 are reported. ns = non-significant difference.

Repertoire size (the number of different tasks a bee performed at least once) did not differ between allogroomers and same age range non-grooming bees (F = 0.102, df = 1.113, p = 0.750, 53 allogroomers vs 62 non-grooming bees) (Fig. 3a). Allogroomers and same age range non-grooming bees did not differ in the performance rate (number of observations scans in which the focal bee performed that behaviour) for any of the behavioural task considered (Table 1, Fig. 3b), unsurprisingly except for the defining behaviour of allogrooming, which was expressed at a significantly higher, even if rather low, frequency by allogroomers (Table 1; median and interquartile range: allogroomers, 0.03, 0; same age range non-grooming bees 0, 0). Moreover, overall activity rates (number of scans during which the bee was performing any behaviour other than inactivity) did not differ between allogroomers and same age range non-grooming bees (F = 0.003, df = 1.113, p = 0.958).

**Table 1:** Behaviours considered in order to describe the behavioural repertoire of same age range allogrooming and non-grooming bees.

Behaviour code	Behaviour category	F	df	p
BC	brood care	0.125	1,113	0.724
SI	social interaction	0.242	1,113	0.624
SG	self-grooming	0.195	1,113	0.660
AG	**allogrooming**	**4.435**	**1,113**	**0.037**
EX	external activity	0.002	1,113	0.964
WL	walking	0.001	1,113	0.976
IN	inactivity	0.005	1,113	0.945
OT	other activities	0.206	1,113	0.607

F, degree of freedom (df) and p values are reported for each comparison.

### Prediction 2. Allogroomers are more central in the colonial social network

**Figure 4:**
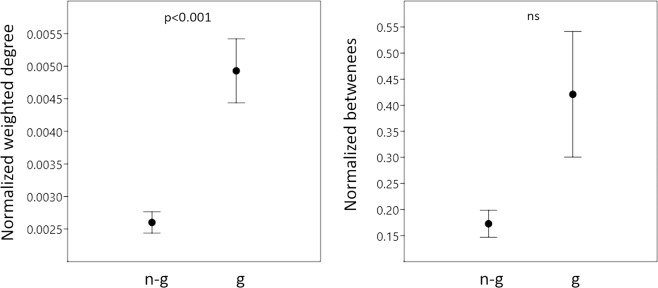
Allogroomers are more central in the colonial social network. Mean and standard error values of centrality (weighted outdegree is the strength of all ties connected to a node, left) and betweenness (the total number of shortest paths between pairs of nodes that pass through the considered node, right) between allogroomers (g, n = 140) and non-groomer bees (n-g, n = 711). ns = non-significant difference.

Allogroomers (n = 140) showed a higher degree centrality (degree centrality is defined as the sum of the strength of all ties connected to a node) than same age range non-grooming bees (n = 711) (Chi-square = 12.452, df = 1, p < 0.001) while they did not show differences in their betweenness (the total number of shortest paths between pairs of nodes that pass through the considered node) (Chi-square = 2.719, df = 1, p = 0.099) (Fig. 4). While colony of origin had a significant effect on both degree (Chi-square = 22.132, df = 1, p < 0.001), and betweenness (Chi-square = 316.401, df = 1, p < 0.001), the interaction between colony of origin and bee category did not have a significant effect on neither degree (Chi-square = 0.557, df = 1, p = 0.456) nor betweenness centrality (Chi-square = 0.032, df = 1, p = 0.858).

### Prediction 3. Allogroomers do not have a differential expression of antennal olfactory proteins compared to same age range non-grooming bees

**Figure 5:**
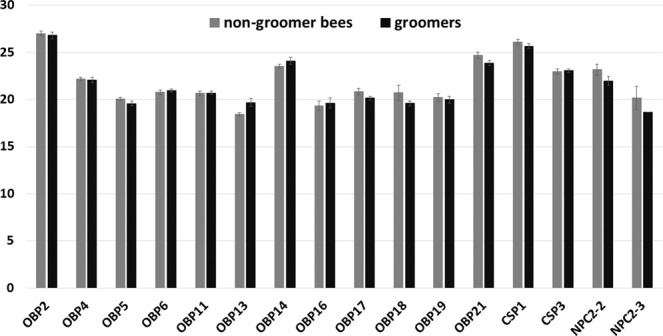
Allogroomers do not have a differential expression of antennal olfactory proteins compared to same age range non-grooming bees. Bar charts showing Log2 LFQ (Label-free quantification) intensities of the identified soluble olfactory proteins (Odorant Binding Proteins, OBP; Chemosensory Proteins, CSP; Niemann-Pick type C2, NPC-2), averaged among the biological replicates of groomers (dark grey bars) and non-groomer bees (light grey bars) respectively. For each sample standard error is also reported. No significant differences were found.

Overall, the “shotgun” approach (i.e. direct digestion of the entire protein extract without a previous separative step) applied on the crude extracts of antennae of same age range non-grooming and allogroomers identified 482 and 484 proteins respectively. The relatively low number of identified proteins depends on the sensitivity of the available equipment when compared to those used in recent studies of insect antennal proteomics. The global distribution of the identified proteins and their expression level are very similar between the two groups (Fig. S1, five biological replicates per group). This high degree of overlap is also reflected considering the numbers of proteins belonging to each gene ontology (GO categories), both for molecular function and for biological process, Pfam and Interpro. Besides the global expression pattern of antennal proteins, our primary aim was to understand if the allogroomers could have a chemosensory specialization based on a different profile of olfactory proteins with respect to same age range non-grooming workers. Among olfactory proteins we identified 12 Odorant Binding Proteins (OBPs), two Chemosensory Proteins (CSPs), and 2 Niemann-Pick type C2 (NPC-2) proteins. OBPs and CSPs are two broad families of insect proteins involved in the first steps of odour recognition^40,41^ by transporting hydrophobic odorants through the sensillar lymph to the odorant-receptors (ORs) borne on the olfactory neurons^42^. A similar role, although less documented, has recently been suggested for NPC-2. None of soluble olfactory proteins is significantly more abundant in allogroomers or same age range non-grooming workers, including CSP3, which has been reported to be associated with grooming by Guarna and coworkers^38,39^ in experimental colonies selected for hygienic behaviour. Therefore, we found no evidence that allogroomers are specialized in the perireceptors events of olfaction (Fig. 5, Fig. S1). Prophenoloxidase, an important component of insect innate immune response also reported to be associated to grooming behaviour^39^, was identified but differences in abundance were not found in our specimens.

No quantitative differences in antennal protein expression between allogroomers and same age range non-grooming workers were found using Mann–Whitney test with a Benjamini-Hochberg correction (FDR ≤ 0.01).

### Prediction 4. Allogroomers have higher immunocompetence compared to same age non-grooming workers

**Figure 6:**
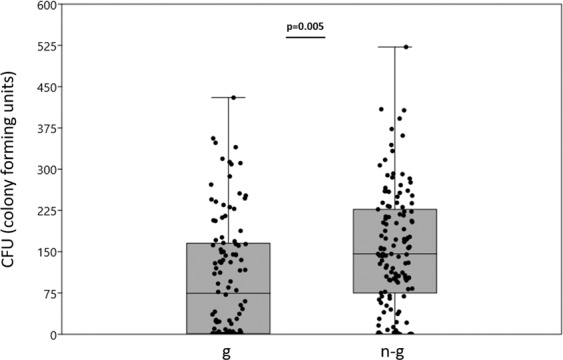
Allogroomers have higher immunocompetence compared to same age range non-grooming workers. Raw numbers of colony forming units (median and quartiles are represented) detected on the agar surface after plating 100 µl of homogenate dilutions in allogroomers (g) and non-groomer bees (n-g). Dots represent the individual values.

Category (allogroomers vs same age range non-grooming workers, respectively n = 108 and n = 127) had a significant effect on immunocompetence (Wald chi-square = 7.752, df = 3, p = 0.005), with allogroomers being more able to clear viable bacterial cells from their hemolymph than same age range non-grooming workers (means ± SE; allogroomers: 103.19 ± 10.47; non-grooming workers: 158.56 ± 18.43) (Fig. 6). Hive of origin did not have a significant effect (Wald chi-square = 2.649, df = 3, p = 0.449). There was no significant interaction between hive of origin and category (Wald chi-square = 0.941, df = 3, p = 0.816). No Colony Forming Units (CFUs) were detected in workers of both categories injected with only PBS.

## Discussion

Through an integrated approach involving behavioural observations and physiological assays, we here demonstrate that allogrooming in the honeybee *Apis mellifera* is a transient behavioural specialization, mirrored by an increased immunocompetence but without a chemosensory specialization in terms of differential abundance of chemosensory proteins in the antennae.

Our observations clearly showed that allogrooming is age-dependent, with a peak of expression between 6 and 11 days of worker age, and its expression is not randomly distributed on the comb surface but rather clustered in a restricted area opposed to the hive entrance and to the dance area. Moreover, our results suggest that allogrooming is a weak specialization. The behavioural repertoire of allogroomers and age-matched non-groomers is quite similar: apart from the defining activity of allogrooming, allogroomers perform the same set of tasks expected on the basis of their age polyethism, and with similar frequency, compared to same age range non-grooming bees. Our results differ from some of previous findings^29,43,44^ in which individual bees performed allogrooming at significant rates for their entire life, without undertaking the typical polyethism path^45,46^. A possible explanation is that variation in allogrooming expression also exists within the category of allogroomers themselves, with a small percentage of individuals showing a hyper-specialization in this task, as already suggested^27^. Our results suggested however that, if this was the case, these bees nonetheless represent a tiny percentage of allogroomers in the colony. Overall, our behavioural investigation, which for the first time specifically compared a large sample of allogroomers and age matched non-grooming bees, allows to characterize allogrooming as a transient and weak behavioural specialization. The finding that behavioural plasticity is maintained by allogroomers despite their specific task is indeed not puzzling in the view of colony task organization^47^. In fact, previous work has shown that the worker temporal polyethism is highly flexible, responding to both genetic influences and colony social requirements^45,47–51^.

Despite the individual behaviour of allogroomers in terms of task performance is similar to that of same age range non-grooming workers, these two categories show significant differences in their position within the colony social network suggesting a spatial specialization in allogroomers. Allogroomers are more connected, i.e. have higher network centrality, compared to same age range non-grooming workers, which translates into contacting a higher number of colony mates. From a proximate perspective, this might be due to a different use of space by groomers. Individual network position has been shown to depend on spatial behaviour in bees^31^. While our results show that they are no more active than non-groomers, they might have less fidelity to specific parts of the comb and/or move faster across the comb. From an ultimate perspective, we might speculate that a higher centrality could be beneficial as it would allow allogroomers to screen a higher number of colony members for parasites. Interestingly, the possible increased costs of such higher network centrality (increased exposure to pathogens^32^) might be reduced in allogroomers thank to their increased levels of immune ability (our results).

The peculiar task of allogroomers might be supported by differential chemosensory abilities that could help in identifying unhealthy individuals to better direct allogrooming towards the most suitable targets inside the colony (prediction 3). Indeed, Guarna and colleagues^39^ reported that Chemosensory protein 3 is linked to grooming behaviour in bees coming from colonies selected and tested for hygienic behaviours. Our results, however, do not support this hypothesis, since at least for soluble olfactory proteins no differences were found when directly comparing allogroomers with same age range non-grooming workers. There are two possible explanations for this finding. First, allogroomers might not need a chemosensory specialization. Bees in need of being groomed can be recognized through other channels, such as the tactile one, or by their behaviour. Indeed, grooming is sometimes solicited through the so called “grooming invitation dance”, whereby bees shake their whole body from side‐to‐side producing specific vibrations which increase the probability of being groomed by a nestmate^52^. Moreover, allogrooming might also be performed on specific age-class individuals rather than on parasitized individuals, and recognition of these individuals may not require a specialized olfaction. Alternatively, we should also recognize that chemosensory abilities do not only depends on the expression of olfactory proteins in the antennae, as the olfactory perception process is a long way from the binding of odorant molecules, at the level of chemosensillar lymph, to the integration in the central nervous system. The absence of substantial differences at the antennal proteome level is thus not, per se, a definitive proof for the absence of chemosensory specialization. Future studies are needed to assess the perceptive abilities of allogroomers at different levels and both using different (such as proboscis extension reflex, electroantennography) and more sensitive techniques. The picture is clearer in bee colonies selected for hygienic behaviour^38^ for which 7 proteins were more expressed in antennae and were considered by authors as protein biomarkers for this specific selective breeding.

While antennal proteome is not peculiar in allogroomers, a clear and striking difference in immunocompetence has been found between allogroomers and same age range non-grooming workers, with the former being more efficient in clearing bacterial cells from their hemolymph. Even if bacterial clearance bioassay represents just one of the possible methods to assess immunocompentence, this technique provides an integrative view of the activation of the organism immune system^53,54^ (and see material and methods section) and the result is thus of great significance. Interestingly, the difference in immunocompetence is even larger with respect to what was previously found when nurse bees and foragers were compared^48,54^. This is particularly intriguing, as allogroomers and non-groomers have the same age and only slightly differ in their behaviour, while nurse bees and foragers largely differ in both age, physiology and behaviour.

Among honeybee workers, social interactions appear to increase towards nestmates showing signs of potential infections^55,56^. Grooming parasitized or sick nestmates to free them from parasites, debris, spores or other infective agents might increase the risk to be infected. Therefore, the enhanced individual immunity showed by allogroomers might contribute to carry out their immunity-related task inside the hive. Our study did not clarify whether the higher immune ability of groomers is part of their specialization toolkit, as it is the case for other physiological specialization in bees, such as increased Juvenile Hormone level in guard bees^57,58^ or rather a consequence of the increased exposure to pathogens due to their increased network centrality. Future studies should address this issue, possibly by directly manipulating individual exposure to pathogens and through non-lethal sampling of hemolymph, in order to follow ontogenetic development of immune ability in allogroomers and non-groomers. Moreover, future studies should also investigate the identity of bees receiving allogrooming. Our finding that the area where allogrooming occurs is opposed to the hive entrance and to the dance area suggests that allogrooming is not directed preferentially toward foragers. However, ad hoc studies are needed to clarify the identity of the receivers of the allogrooming acts.

A possible limitation of our study is that, in order to obtain a relatively large number of allogroomers, we were not able to continuously follow individual worker behaviour for a long period. This could have led us to include in the sampling a mix of bees with variable levels of specialization in allogrooming. For example, it is not possible to exclude that proteomic differences might emerge if only hyper-specialized groomers were compared with groups of bees that have never shown allogrooming for their whole life (a possibility prevented by our non-continuous behavioural sampling). However, our approach allowed to show that, under our working definition of allogroomers and non-groomers, and given our sampling scheme, striking phenotypic differences between the two groups emerge in some phenotypic aspects of both behaviour (position within the social network) and physiology (immunocompetence) but not in other ones (task performance and antennal proteomic profile).

The implications of our study are twofold. Firstly, we provide evidence to better understand the degree of specialization of allogrooming in *A. mellifera* which appears as a weak and transient behavioural specialization, characterized by a marked enhanced immunity. Since the expression rate of the behaviour appears to be limited also at the colony level in terms of individuals performing the behaviour with respect the total number of individuals, we can suppose that allogrooming is expressed in *A. mellifera* but not to an extent that could effectively contrast the parasite pressure of *Varroa destructor*^59–61^. Although this evidence might be discouraging, strategies to increase the overall colony rate of allogrooming in order to cope with this very impacting pest might still be considered as reasonable. The second insight from our results is that the physiological specialization of allogroomers is specifically related to the immune phenotype. Our results indicate an enhanced immune response in allogroomers compared to the same age range non-groomers nestmates as well as a different position of allogroomers in the colonial social network, in agreement with predictions from organizational immunity^31^.

Overall, our results demonstrate the presence of an immune-specific physiological and social behaviour specialization in individuals involved in social immunity related tasks, thus linking individual to social immunity. It also suggests that division of labour might lead to physiological specialization narrowly tailored upon the task performed while maintaining an overall plasticity.

## Material and Methods

### Insect collection, rearing and general procedures

Experiments were conducted between June and August, when expression of allogrooming is higher^22^ and pers. obs., in 2014 (observations for individual behaviour characterization), 2015 (antennal proteomics and bacterial challenge) and 2016 (behavioural observation for social network analysis). All studies were performed using standard one-frame observation hives maintained in laboratory (Department of Biology, University of Florence) where bees were free to forage outside. Observation combs were taken from colonies belonging to a local beekeeper (Apicoltura Cristofori Mauro). We screened 15 colonies each year in order to identify colonies with significant allogrooming rates (at least 30 allogrooming acts per comb during 30 min of observation). From each selected colony we took a comb containing stored honey and pollen, open and sealed brood cells, the queen and circa 2000 workers and transferred it to the observation frame. Experiments were performed on four observation hives for the behavioural experiments (two for prediction 1 and two for prediction 2), three hives for antennal proteomics (prediction 3) and four observation hives for the bacterial challenge (prediction 4). Overall, observation combs were issued from a total of 11 colonies.

### Individual bee marking procedure

To obtain house bees of known age needed for the experiments, we collected newly emerged bees directly from the comb by gently removing them with forceps. Bees were a) individually marked with plastic coloured numbered tags on their thorax (predictions 1 and 2, for which we needed to follow individual behaviour over several days) or b) marked with a spot on the thorax with UniPosca® paint markers, using different colours according to day of collection and hive of origin (predictions 3 and 4, where individual age and colony of origin were sufficient information as identified bees were immediately removed from the comb as soon as they performed the grooming act). Marked bees were gathered in plastic cylindrical containers (Ø 10 cm × h 10 cm), and lightly dusted in icing sugar before being gently reintroduced in their natal colonies within a few hours to favour acceptance by older nestmates. More than 200 newly emerged workers per observation comb were marked for testing predictions 1 and 2 and more than 100 per comb for testing predictions 3 and 4 (approximately a total of 1500 bees were marked in total).

### Temporal and spatial dynamics of allogrooming expression

We evaluated the temporal dynamics of allogrooming expression along individual worker lifespan using an all occurrences sampling method, focusing on allogrooming^62^. Starting the day after re-introduction of newly emerged marked bees and until day 25 post emergence (average life expectancy during summer^63^), we counted the occurrences of all allogrooming (Supplementary Video 1) events performed by a marked bee by observing combs every odd day for 30 min at each side. A total of 12 h of observation was performed on each hive. Departure from randomness of the temporal expression of allogrooming (number of bees performing an act of allogrooming on the total number of marked bees) was tested with a Runs test. On average 104.60 ± 19.31 marked bees were observed for each age interval.

We evaluated the spatial occurrence of allogrooming expression on the comb surface by using an all occurrences sampling method, as above^62^. We recorded the spatial coordinates of all allogrooming events performed by any bee by observing three observation combs. We alternated observation of 30 min for each comb side. A total of about 15 h of observation was performed (5.16 h on average for each hive). For each allogrooming act, the position was precisely marked on a transparent acetate sheet overlaid onto the observation frame glass surface. Once observations were collected, their coordinates were pooled across the three combs and departure from complete spatial randomness in distribution of the expression of allogrooming was tested by computing the routine Kest (Ripley’s K-function) and simulating 99% significance envelopes using the envelope function in spatstat package in R^64^. Observations took place during central hours of the day (between 11 am and 4 pm)^21^.

### Prediction 1. Allogroomers show a specific behavioural repertoire

We determined the behavioural repertoire of allogroomers and same age range non-grooming bees using focal animal sampling^62^. Every marked bee observed performing an act of allogrooming (Supplementary Video 1) continuously for at least 30 s (during the morning observation) was followed continuously for 10 min later on during the afternoon of the same day (between 2 and 5 pm). Ten-minute observation periods were divided into 20 intervals of 30 s each, and for each interval the main behaviour performed by the focal bee was recorded, thus obtaining 20 observation scans for each focal groomer. During each ten min period only a single bee (focal bee) was observed. The same procedure was applied to non-grooming bees, i.e. marked bees of the same age range as focal allogroomers never observed to perform acts of allogrooming during the same or previous days. In case bees chosen as same age range non-groomers were later observed performing allogrooming, they were removed from the dataset. This procedure allowed the comparison of behavioural specialization between allogroomers and same age range non-grooming bees at each age. Overall, we obtained 53 focal allogroomers (first hive, n = 26; second hive, n = 27) and 62 focal same age range non-grooming bees (first hive: 26; second hive = 36) ranging in age between 4 and 15 days post-emergence. Age distribution was not different between the two groups (Mann-Whitney test, U = 1414, p = 0.201, N = 53 vs 62). For each bee we calculated: *a)* the behavioural repertoire size as the number of different tasks (listed in Table 1) performed at least once; this measure has been used as a proxy of behavioural plasticity, i.e. the opposite to behavioural specialization^65,66^; *b)* the performance rate of each behavioural item, as the number of observation scans in which the focal bee performed that behaviour.

As many behavioural items were very rarely performed, we pooled them within the same category. Social interactions included antennation, trophallaxis and waggle dance, brood care included larval inspection, larval care and ventilation, the category “other” included comb building and related activities. Moreover, we controlled for possible differences in the behavioural repertoire due to different activity rates, by comparing activity rate (number of scans during which the bee was performing any behaviour other than inactivity) between allogroomers and same age range non-grooming bees. Behavioural repertoire size and performance rates for each behavioural item were compared between the two groups using a GLMM with bee category (allogroomers or non-allogroomers) as fixed factor, hive of origin as random factor and including their interaction. We used a negative binomial distribution for all comparisons, except for three behavioural categories (allogrooming, external activity and other activities) which were performed so rarely that we dichotomize the data and used a binomial logistic distribution. Statistical analyses were performed with SPSS 16.0 (SPSS Inc., Chicago, IL) and Past v3.20^67^.

### Prediction 2. Allogroomers are more central in the colonial social network

An association network was built on the basis of the spatial proximity of bees, following the protocol in Baracchi and Cini^31^. We considered two bees interacting when they were at a distance shorter of the length of a bee body (approximately less than 3 cells). Interaction among marked bees were recorded from each observation colony taking photos, over a period of four days. 25 Photos were taken on both sides every hour during the central hours of the day (approximately 11 am-4 pm). In order to define the association network, each individual bee was considered as a node and an interaction between two individuals was taken as an edge existing between these two nodes, thus resulting in a weighted and not directed network. The following measures of centrality were calculated for each node: degree and betweenness^68^. Degree is defined as the sum of the strength of all ties connected to a node; betweenness is a centrality measure that is defined as the total number of shortest paths between pairs of nodes that pass through the considered node. It is thus an index of liaising otherwise separate parts of the network. We focused on these two measures as they catch different aspects of network centrality, respectively the size of the social neighbourhood of a bee, i.e. the individual potential to groom many bees (degree), and the potential to influence the passage of pathogens through the network (betweenness). Both these measures have shown to differ according to age and task in *A. mellifera*^31^. Centrality measures were computed using Ucinet 6 and differences in centrality measures between allogroomers and same age range non-grooming bees were assessed using a generalized linear model (GLZ) with negative binomial distribution and log-link function with 10000 permutations to take into account the non-independence of network data. The full factorial model included hive of origin as a random factor and category (allogroomers, n = 140; same age range non-grooming bees, n = 711) as a fixed factor as well as their interaction. Statistical analyses were performed with Ucinet 6^69^.

### Prediction 3. Allogroomers have a differential expression of antennal olfactory proteins compared to same age range non-grooming bees

Allogroomers and same age range non-grooming bees (defined as above) were collected from 3 observation hives. As this same study shows allogrooming is mainly performed by bees within a well-defined age-interval (Fig. 2a) we sampled marked bees within this range. Once identified as allogroomers, bees were gently removed from the comb with forceps and immediately stored at −20 °C. For each allogroomer sampled a non-allogroomer of the same age range was collected from the same hive. Non-allogroomers were defined as those workers marked on the same day of the collected allogroomer which have never been observed performing allogrooming during the same or previous days. Then, flagella from antennae were dissected immediately before protein extractions. Extracts were prepared from pools of 5 individuals randomly sampled from the three colonies. Five biological replicates for each group (allogroomers and non-grooming bees) were prepared. Reagents and procedures used for protein extraction, digestion, purification and shotgun analysis (i.e. direct digestion of the entire protein extract without a previous separative step), as well as protein identification and quantification are described in Iovinella *et al*.^37^ (see also Supplementary Material). Protein extracts were reduced, alkylated and digested prior to HPLC-MS analysis. The peptide mixture of each sample was submitted to a nanoLC-nanoESI-MS/MS analysis on an Ultimate 3000 HPLC (Dionex, San Donato Milanese, Milano, Italy) coupled to a LTQ-Orbitrap mass spectrometer (Thermo Fisher, Bremen, Germany). The mass spectrometry proteomics data have been deposited to the ProteomeXchange Consortium via the PRIDE^70^ partner repository with the dataset identifier PXD017651. Data were searched using MaxQuant software (version 1.5.2.6) against databases downloaded from Uniprot containing all *Apis mellifera* proteins as well as those from common honeybee viruses. The data relative to identification and quantification are contained in the MaxQuant output file named proteinGroups.txt and are reported in Supplementary Dataset. Search results were analyzed with the Perseus software platform (version 1.5.1.6) and IBM SPSS v20. Differential protein abundance was evaluated after filtering data for proteins quantified in at least 3 replicates (out of the 10). Hierarchical clustering analyses were performed using average Euclidean distance and the default parameters of Perseus (300 clusters, maximum 10 iterations). Missing values were imputed (width = 0.3, downshift = 1.8) by drawing random numbers from a normal distribution to simulate signals from low abundant proteins, using the default parameters. Analysis of differential abundance of single proteins was performed using Mann–Whitney test, with a Montecarlo resampling procedure (100000 samples), followed by a Benjamini-Hochberg correction (FDR ≤ 0.01), as a substantial fraction of protein abundance values were not normally distributed.

### Prediction 4. Allogroomers have higher immunocompetence compared to same age range non-grooming workers

Allogroomers and same age range non-grooming bees were collected from 4 observation hives following the same criteria used to sample bees for antennal proteome analysis (see prediction 3). We compared the ability to clear bacterial cells from their haemolymph (i.e. bacterial clearance) between allogroomers and same age range non-grooming bees by injecting bees with the Gram-negative bacteria *Escherichia coli*, an immune elicitor commonly used to test immunocompetence in insects^71–74^. We measured bacterial clearance as a good proxy of workers immunity strength since injection of live bacteria provides an integrative view of the activation of the organism immune system^53,54^ and different parameters used to measure antimicrobial immune response in insects are positively correlated^75,76^. Moreover, *E. coli* is not naturally found in *A. mellifera*, we could therefore exclude its presence in our workers prior to artificial infection. Bacterial culture and injection were carried out following the same procedure used by Cappa *et al*.^54^ (see also supplementary material). Each worker (allogroomers, n = 108; same age range non-grooming bees, n = 127) was infected by injecting 1 µl of inoculum, containing approximately 1.5 × 10^5^ cells, with a HamiltonTM (Bonaduz, Switzerland) micro syringe between the second and third tergites. After injection, bees were introduced in groups of about 10-20, separated for category (allogroomers and same age range non-grooming bees), into plastic cylindrical containers (Ø 10 cm x h 10 cm) provided with *ad libitum* honey as food and maintained under controlled conditions (~30 °C; 55% RH). All containers used to house infected bees were carefully washed with ethanol and dried before use to prevent any contamination and then randomly allocated to the allogroomers or same age range non grooming bees. Twenty-four hours later, each worker was quickly beheaded with scissors, and then the whole body was inserted in a sterile plastic bag with 10 mL of PBS after removing the sting and the venom sac to prevent a reduction of bacterial cells viability due to venom antimicrobial peptides^77^ and processed with a Stomacher® 400 Circulator (230 rpm × 10 min) to homogenize the bee body. Afterwards, 0.1 mL of undiluted and serially diluted PBS suspensions (dilutions 10^−1^, 10^−2^) of each sample were plated on LB solid medium added with tetracycline (10 μg/mL) and incubated overnight at 37 °C. The following day, the colonies grown on the plate surface were counted and the viable bacterial count was expressed as Colony Forming Units (CFUs) per bee. At least 8 same age range bees per colony for each category (4 allogroomers and 4 same age range non-grooming bees) were injected with 1 µL of PBS, homogenized and plated following the same procedure of *E. coli*-infected workers, to ensure absence of other bacterial strains capable of growing on our LB agar plates added with tetracycline. A total of 235 bees were infected with *E. coli* and plated: (i) allogroomers, n = 108, (ii) same age range non-grooming bees, n = 127. Bacterial challenge data (raw number of CFU) were analyzed with a generalized linear model (GLZ) with negative binomial distribution and log-link function. The full factorial model included hive of origin as a random factor and category (allogroomers vs non-grooming bees) as a fixed factor. Statistical analyses were performed with SPSS 16.0 (SPSS Inc., Chicago, IL) and Past v3.20^67^.

## Supplementary information

Supplementary Information.

Supplementary Dataset S1.

## Data Availability

Proteomic data are available via ProteomeXchange with identifier PXD017651. The other datasets generated and/or analysed during the current study are available in Supplementary materials and/or from the corresponding author on reasonable request.
