# Supplementary material for: Increased immunocompetence and network centrality of allogroomer workers suggest a link between individual and social immunity in honeybees
Source: Sci Rep. 2020 Jun 2;10:8928. doi: 10.1038/s41598-020-65780-w (PMC7265547; doi:10.1038/s41598-020-65780-w)
Supplement: Supplementary file 1 — Supplementary Information. [file 41598_2020_65780_MOESM1_ESM.docx]

**Supplementary material**

**Increased immunocompetence and network centrality of allogroomer workers suggest a link between individual and social immunity in honeybees**

Alessandro Cini^1,2,#^, Adele Bordoni^1^, Federico Cappa^1^, Iacopo Petrocelli^1^, Martina Pitzalis^1^, Immacolata Iovinella^1^, Francesca Romana Dani^1,3^, Stefano Turillazzi^1^, Rita Cervo^1^

^1^ Dipartimento di Biologia, Università di Firenze, Via Madonna del Piano 6, 50019, Sesto Fiorentino, Firenze, Italy

^2^ Centre for Biodiversity and Environment Research, University College London, Gower Street, London WC1E 6BT

^3^ CISM, Mass Spectrometry Centre, University of Firenze, Via U. Schiff 6, 50019 Sesto Fiorentino, Italy

^#^ corresponding author: Alessandro Cini, [cini.ales@gmail.com](mailto:cini.ales@gmail.com)

ORCID: [0000-0003-0355-218](https://orcid.org/0000-0003-0355-2188)

**Material and methods**

*Detailed protocol for antennal proteome characterization*

Reagents

Ammonium bicarbonate, DTT, iodoacetamide, sodium chloride, formic acid, acetonitrile, trifluoroacetic acid, acetic acid, thiourea were from Sigma-Aldrich (Milano, Italy), while Tris and urea from Euroclone. Trypsin was purchased from Promega (Sequencing Grade Modified Trypsin) and Lys-C from Thermo Scientific (MS grade). The hand-made desalting/purification STAGE column were prepared using three C18 Empore Extraction Disks (3M).

Protein Sample Preparation and Digestion

Antennae were dissected and pooled immediately before protein extractions from pool of 5 individuals. Five biological replicates for each sample were prepared. The extracts were prepared crushing the tissue in a mortar under liquid nitrogen and the proteins extracted with 6 M Urea/2 M Thiourea in Tris-Cl 50 mM pH 7.4. The protein extracts were centrifuged at 14000 rpm for 40 min at 4 °C and the supernatants were collected for the analysis. The total amount of protein in each sample was assessed by the Bradford colorimetric assay, with the “Bio-Rad Protein Assay” kit using serial dilutions of bovine serum albumin to generate a standard curve. Protein sample concentration was measured by Infinite PRO 200 reader (TECAN).

Protein digestion was carried out on 15 μg protein extracts. Reduction of disulfide bridges was performed by treating samples with DTT (1 μg of DTT/50 μg of proteins for 30 min at RT), followed by alkylation (5 μg of iodoacetamide/ 50 μg of proteins for 20 min at RT in the dark), as described by Foster and co-workers (Foster et al., 2003). Protein samples were diluted 3 times with 500 mM ammonium bicarbonate, to increase pH and reduce the concentration of urea/thiourea. A first enzimatic digestion was performed by incubating the samples with LysC in a ratio 1:50 (w/w) for 3 h at 37 °C. The digestion products were then incubated with trypsin in a ratio 1:50 (w/w) overnight at 37 °C. The digested samples were then acidified by adding trifluoracetic acid and desalted on STop And Go Extraction (STAGE) tips (Rappsilber et al., 2007). The eluates were concentrated and reconstituted to 20 μL in 0.5% acetic acid, prior to HPLC-MS analyses.

Mass Spectrometric Analysis

The peptide mixture of each sample was submitted to a nanoLC-nanoESI-MS/MS analysis on an Ultimate 3000 HPLC (Dionex, San Donato Milanese, Milano, Italy) coupled to a LTQ-Orbitrap mass spectrometer (Thermo Fisher, Bremen, Germany). Fractions containing 2.25 μg protein were injected directly on a homemade nano column packed with Aeris Peptide XB-C18 phase (75 μm i.d. × 15 cm, 3.6 μm, 100 Å, Phenomenex, Torrance, CA, USA) and eluted with a flow rate of 300 nl/min. The elution mobile phases composition was: H_2_O 0.1% formic acid/CH_3_CN 97/3 (phase A) and CH_3_CN 0.1 % formic acid/H_2_O 80/20 (phase B). The elution program was: 0 min, 2% B; 40 min, 2% B; 68 min, 15% B; 168 min, 25% B; 228 min, 35% B; 273 min, 50% B; 274 min, 90% B; 288 min, 90% B; 289 min, 2% B; 309 min, 2% B. Mass spectra were acquired in positive ion mode, setting the spray voltage at 1.8 kV, the capillary voltage and temperature respectively at 45 V and 200 °C, and the tube lens at 130 V. Data were acquired in data dependent mode with dynamic exclusion enabled (repeat count 2, repeat duration 15 s, exclusion duration 30 s); survey MS scans were recorded in the Orbitrap analyzer in the mass range 300-2000 m/z at a 15,000 nominal resolution at m/z = 400; then up to five most intense ions in each full MS scan were fragmented (isolation width 3 m/z, normalized collision energy 30) and analyzed in the IT analyzer. Monocharged ions did not trigger MS/MS experiments.

Data processing

The identification of proteins was performed using MaxQuant software (version 1.5.2.6) (Cox and Mann, 2008). The derived peak list was searched with Andromeda search engine (Cox et al., 2011). We used as database all the proteins of *Apis mellifera* from Uniprot merged with a set of commonly observed contaminants, such as human keratins, bovine serum proteins, and proteases. Data were also searched against databases from common honeybee viruses, downloaded from Uniprot. In parameter section, we set as enzyme Trypsin and Lys-C, allowing up to two missed cleavages. The minimum required peptide length was seven amino acids. Carbamido-methylation of cysteine and oxidation of methionine were set as variable modifications. As no labeling was performed, multiplicity was set to 1. During the main search, parent masses were allowed an initial mass deviation of 4.5 ppm and fragment ions were allowed a mass deviation of 0.5 Da. PSM (Peptide Spectrum Match) and protein identifications were filtered using a target-decoy approach at a false discovery rate (FDR) of 1%.

Relative, label-free quantification (LFQ) of proteins was done using the MaxLFQ algorithm integrated into MaxQuant. The match between runs option was enabled with a match time window of 4 min and an alignment time window of 20 min. For protein quantification we used 1 as Minimum ratio count, “Unique+Razor” peptides (i.e. those exclusively shared by the proteins of the same group), peptides with variable modifications, and selected “discard unmodified counterpart peptide”.

Annotations regarding gene onthology (GO) categories, Protein family (Pfam) and Interpro were downloaded from the link (http://141.61.102.106:8080/share.cgi?ssid=0q4b6sT) available in Perseus software (version 1.5.1.6) and each protein identifier was associated with these categories if available. The data were filtered to eliminate hits to the reverse database, contaminants and proteins only identified with modified peptides.

References

[Foster LJ](http://www.ncbi.nlm.nih.gov/pubmed/?term=Foster%20LJ%5BAuthor%5D&cauthor=true&cauthor_uid=12724530), [De Hoog CL](http://www.ncbi.nlm.nih.gov/pubmed/?term=De%20Hoog%20CL%5BAuthor%5D&cauthor=true&cauthor_uid=12724530), [Mann M](http://www.ncbi.nlm.nih.gov/pubmed/?term=Mann%20M%5BAuthor%5D&cauthor=true&cauthor_uid=12724530). Unbiased quantitative proteomics of lipid rafts reveals high specificity for signaling factors. [Proc Natl Acad Sci U S A.](http://www.ncbi.nlm.nih.gov/pubmed/12724530) 2003;100:5813-8.

Rappsilber J, Mann M, Ishihama Y. Protocol for micro-purification, enrichment, pre-fractionation and storage of peptides for proteomics using StageTips. Nat Protoc. 2007;2:1896-906.

Cox J, Mann M. MaxQuant enables high peptide identification rates, individualized p.p.b.-range mass accuracies and proteome-wide protein quantification. Nat. Biotechnol. 2008;26:1367–1372.

Cox J., Neuhauser N., Michalski A., Scheltema R. A., Olsen J. V., Mann M. Andromeda: a peptide search engine integrated into the MaxQuant environment. J. Proteome Res. 2011;10:1794–1805.

*Detailed protocol for bacterial clearance assays*

Bacterial culture and artificial infection

Bacterial cultures of *E. coli* tetracycline-resistant strain XL1 Blue (Stratagene, La Jolla, CA, USA) were grown aerobically in Luria-Bertani (LB) complex medium containing tetracycline at a concentration of 10 μg/mL overnight at 37 °C in a shaking incubator. After centrifugation, bacteria were washed twice in phosphate-buffered saline (PBS), resuspended and diluted to the desired concentration with PBS (~1.5 x 108 cells/ml). The approximate amount of bacterial cells in the solution was determined using a haemocytometer (Neubar) and confirmed by plating the bacterial solution on LB agar (dilutions 10^-6^, 10^-7^) and counting the colony forming units (CFUs) that grew overnight at 37 °C. Each bee was infected by injecting 1 µL of inoculum, containing approximately 1.5 x 105 cells, with a HamiltonTM micro syringe between the 2^nd^ and the 3^rd^ tergite. Before injection, workers were cooled down in a refrigerator (T 8°C) to facilitate their manipulation. After infection, bees were introduced in groups of about 10-20, separated for category, into plastic cylindrical containers (Ø 10 cm x h 10 cm) provided with *ad libitum* honey inside pearced Petri dishes (Ø 2.5 cm) as food. Bees were then maintained under controlled conditions (approximately 30 °C; 55% RH) for 24 h until subsequent homogenization and plating.

**Results**


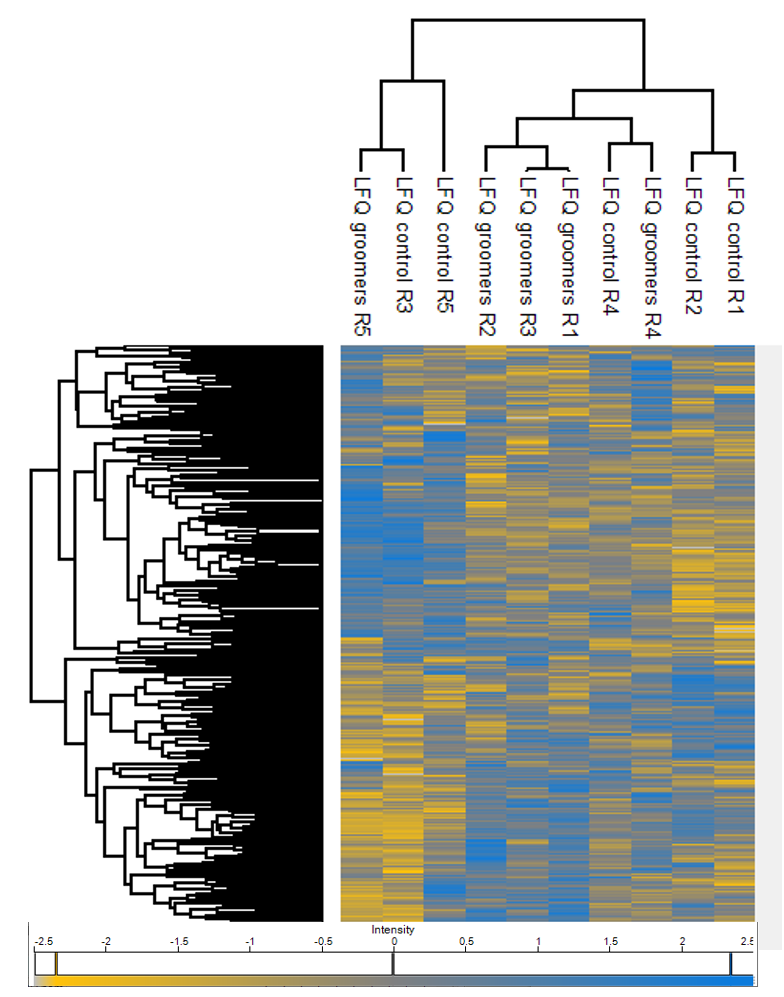


**Supplementary Figure S1.** Heatmap representation of the expression of single protein levels between groomers and control bees. The map has been built making an unsupervised hierarchical clustering (300 clusters, maximum 10 iterations) based on LFQ (Label-free quantification) values of proteins with at least 3 observations (out of 10). Colour scale reports Z-score Log2 transformed LFQ intensity values. Missing data are reported in grey. No differences have been highlighted between the two groups as displayed in the cluster grouping biological replicates.

**Supplementary dataset S1.** Complete list of proteins identified in proteomic analysis of antennae of *Apis mellifera* groomers and non-groomers. The table contains information on the proteins identified in all processed raw-files. Each single row contains the group of proteins that could be reconstructed from a set of peptides.
